# French Public Familiarity and Attitudes toward Clinical Research during the COVID-19 Pandemic

**DOI:** 10.3390/ijerph18052611

**Published:** 2021-03-05

**Authors:** Émilien Schultz, Jeremy K. Ward, Laëtitia Atlani-Duault, Seth M. Holmes, Julien Mancini

**Affiliations:** 1 CEPED (UMR 196), Université de Paris, IRD, 75006 Paris, France; laetitia.atlani-duault@ird.fr; 2 SESSTIM, Sciences Economiques & Sociales de la Santé & Traitement de l’Information Médicale, CANBIOS Team (Équipe Labellisée LIGUE 2019), Aix-Marseille University, INSERM, IRD, 13009 Marseille, France; sethmholmes@berkeley.edu (S.M.H.); julien.mancini@inserm.fr (J.M.); 3 CERMES3, INSERM, CNRS, EHESS, Université de Paris, 94801 Villejuif, France; jeremy.ward.socio@gmail.com; 4 VITROME, Aix-Marseille University, IRD, AP-HM, SSA, 13005 Marseille, France; 5 Institut COVID-19 Add Memoriam, University of Paris, 75006 Paris, France; 6 WHO Collaborative Center for Research on Health and Humanitarian Policies and Practices, IRD, Université de Paris, 75006 Paris, France; 7 Society and Environment, Medical Anthropology, and Public Health, University of Berkeley, Berkeley, CA 94720, USA; 8 Mediterranean Institute for Advanced Study IMéRA, Institut Paoli Calmettes, Aix-Marseille University, 13004 Marseille, France; 9 BioSTIC, APHM, Timone, 13005 Marseille, France

**Keywords:** clinical research, public attitudes, survey, COVID-19, pharmaceutical industry, media coverage

## Abstract

The COVID-19 pandemic put clinical research in the media spotlight globally. This article proposes a first measure of familiarity with and attitude toward clinical research in France. Drawing from the “Health Literacy Survey 2019” (HLS19) conducted online between 27 May and 5 June 2020 on a sample of the French adult population (N = 1003), we show that a significant proportion of the French population claimed some familiarity with clinical trials (64.8%) and had positive attitudes (72%) toward them. One of the important findings of this study is that positive attitudes toward clinical research exist side by side with a strong distancing from the pharmaceutical industry. While respondents acknowledged that the pharmaceutical industry plays an important role in clinical research (68.3%), only one-quarter indicated that they trust the industry (25.7%). Positive attitudes toward clinical trials were associated with familiarity with clinical trials (Odds Ratio, OR 2.97 [1.90–4.63]), financial difficulties (OR 0.63 [0.46–0.85]), as well as mistrust of doctors (0.48 [0.27–0.85]) and of scientists (OR 0.62 [0.38–0.99]). Although the French media provided a great deal of information on how clinical research works during the first months of the pandemic, there remains profound mistrust of the pharmaceutical industry in France. This suspicion can undermine crisis management, especially in the areas of vaccine development and preparation for future pandemics.

## 1. Introduction

During the COVID-19 pandemic, the urge to identify a treatment or vaccine boosted clinical research worldwide [1]. New trials and scientific publications received intense media coverage and fierce debates arose on such issues as hydroxychloroquine efficacy [2]. This put a magnifying glass on the organisation of medical research, with harsh criticisms leveled against the influence of the pharmaceutical industry and the medical elite over health policy [3]. This polemical situation had a direct impact on public participation in clinical trials, including by reducing patient enrollment in the non-treatment arms of trials on hydroxychloroquine [4]. Because news coverage impacts the whole society [5], it is also likely to have long-term consequences on people’s attitudes toward clinical research and health authorities.

Since the middle of the 20th century, clinical trials conducted through collaboration between the public and private sectors have been a crucial step in the process of medical innovation. They came to be perceived as beneficial to enrolled patients both by patients themselves and by the general public [6,7]. Over time, their increasing importance raised new issues—e.g., their mode of implementation [8,9]—new ethical concerns—e.g., the “therapeutic misconception” whereby patients perceive only the immediate personal therapeutic promise of a clinical trial [10]- and new challenges—e.g., patients’ new experiences of care in the context of a clinical trial [11,12]. At the same time, public attitudes toward the pharmaceutical industry became increasingly negative in many countries, perhaps especially in France [13], where discourses against “Big Pharma” began to take hold in many aspects of society [14], especially around the topic of clinical trials [15]. Moreover, attitudes toward physicians have deteriorated in some populations due to their being associated with the private sector [16,17]. This has been an increasing source of concern for the medical profession in recent years [3,18].

It is then important to understand public attitudes towards clinical research more thoroughly, as these affect the organisation of clinical trials and, more generally, public acceptance of and implementation of recommendations for preventive behavior change along with public trust in health policy. Few studies have examined this topic to date. Available studies have focused mainly on specific areas of research such as HIV or cancer [19,20] and assessed the factors that influence patients’ participation in clinical trials, such as education [21], their perception of clinical trials [22], or their trust in doctors [23]. A recent international survey focused on public awareness and knowledge of clinical trials conducted in 68 countries: this study found positive attitudes toward clinical research and a general willingness to participate in clinical trials, with a significant age effect [24,25]. However, both studies focused on patients’ willingness to enroll and large scale studies based on convenience sample lack national representativeness and do not engage crucial national trends, such as growing defiance toward the private sector in France. Furthermore, because of local idiosyncrasies in healthcare systems and public debates on health-related issues [26], focusing on specific national cases can help identify more precisely the conditions allowing for widespread acceptance of clinical trials or the emergence of public debates such as those surrounding vaccines in the context of rising vaccine hesitance in the past ten years in France [27].

This study carried out on a sample of the French population during the COVID-19 outbreak offers a first examination of the French public’s familiarity with and attitudes toward clinical research and clinical trials. It draws on insights from the social sciences to explore these trends and their main determining factors. Health literacy has been shown to impact the attitudes toward clinical trials [21]. We expected that health literacy and information seeking behaviors would have an effect on familiarity [20]. As attitudes toward clinical research may relate to broader attitudes toward science, we also expect that trust in institutions, doctors and scientists, would play an important role [28]. We also test the influence of other factors such as demographic factors and attitudes toward the severity of the COVID-19 pandemic, considering the fact that the context of the crisis created a specific debate in France on clinical research [29]. In doing so, our study contributes to informing public health debates on therapeutic innovation and the importance of clinical research in the management of crises, bridging questions related to health choices and public understanding of the health sciences [30,31].

## 2. Materials and Methods

### 2.1. Design and Sample

The data analysed in this paper were collected via the French “Health Literacy Survey 2019” (HLS19). We conducted a cross-sectional online survey among a sample of the French population aged 18–75 (N = 1003) two weeks after the end of the full lockdown in France (between 27 May and 5 June 2020). Participants were selected from an online nationally representative research panel of households of the French general population developed and maintained by the survey research firm IPSOS (Paris, France). A total of 23,289 individuals were initially invited by mail to fill out the survey. Quota sampling was managed to match French official census statistics for gender, age, size of the population in the area of residence and region. The largest difference between theoretical quotas and our sample was −1.5% for 18–25 years old participants (11.5% of the sample instead of the 13% planned). Collected data were then weighed according to the respondent’s demographic profil to match the national distribution regarding those four dimensions. The study was approved by the Ethics Evaluation Committee of the French national biomedical research Institute INSEM (CEEI, IRB 00003888, 202/04/04).

### 2.2. Data Collection

After obtaining informed consent, respondents answered a self-administered online questionnaire broaching five main themes: (1) demographic characteristics (gender, age, level of education, region of residence, financial difficulties, occupation, current health condition); (2) health literacy, ability to navigate the healthcare system and communication with caregivers through new technologies; (3) perception of and familiarity with clinical research; (4) trust in institutions; and (5) knowledge and concerns about the coronavirus epidemic.

The items on clinical research focused on familiarity with and attitudes toward clinical trials, but also on perceptions of clinical trials’ therapeutic usefulness and of the role they play in health care and knowledge production (Table 1). These items were developed and tested in previous research [21].

In this article, we used two main questions to assess, respectively, a 5-point scale for familiarity and a 10-point scale for attitude. The first question asked “How familiar are you, if at all, with clinical trials?” with answers ranging from 1 “Never heard of clinical trials before today”, 2 “I only know the term clinical trial”, 3 “Not very familiar with what clinical trials are”, 4 “Quite familiar with what clinical trials are” to 5 “Extremely familiar with what clinical trials are”. The second question asked “Based on what you currently know, what is your overall impression of clinical trials ?” with a score ranging from 1 “Very Negative Impression” to 10 “Very Positive Impression”. These questions were supplemented with more detailed questions on clinical research and clinical trials (Table 1). For Q-B-6, we aimed to assess perceptions of the specificity of research on COVID-19: half of the respondents were asked to answer a question on the specifics of biomedical research on cancer and the other half were asked to answer a question on the specifics of biomedical research on COVID-19 (questions were distributed at random). Items pertaining to trust (in scientists, doctors, politicians and industry) were drawn from the literature on public perception of science in France [30].

### 2.3. Statistical Analysis

Several variables were recoded both to enhance comparability, interpretability and improve group counts: “Educational level” was recoded into three groups according to whether participants had completed high school; “age” was recoded into four groups. Four-point scales were dichotomized into two modalities (“Yes” and “No”): “trust in doctors, politicians, scientists and industry”, “concern about COVID”.

We used a construct for financial difficulties coding “yes” if they declared to have difficulties to pay bills by the end of the month or to pay for medical examinations and treatments in case of need.

We used a preexisting construct for actual Health literacy [32], self-assessed using the 16-item version of the European Health Literacy Scale (HLS-EU). Each item of this scale was dichotomized into levels “easy” (value 1) and “difficult” (value 0) and the score was calculated by summing up the values obtained for each participant. Three categories were generated according to the health literacy score: Inadequate (HLS-EU < 9), Problematic (HLS-EU [9–12]) and Adequate (HLS-EU > 12).

We measured association between variables with the r Pearson’s correlation coefficient for numeric variables and $\chi^{2}$ statistics for categorical variables. Even if the sample is not randomly selected, we used the pooling institute indicative error margins for the interpretation of the estimated proportions, ranging from 1.4 to 3.1 points (depending of the value of the proportion).

Two binomial logistic regression analyses were performed to identify the main adjusted factors influencing the reporting of high familiarity with and positive attitudes toward clinical trials. First, we dichotomized the two variables: a respondent was coded as “familiar” if he/she answered as “Quite” (4) or “Extremely” (5) familiar with what clinical trials are. He/she was considered having a positive attitude of clinical trials if he/she answered 6 or more for their impression of clinical trials. We then used a purposeful selection of the variables to establish a main effect model [33], first selecting in the binomial regression variables displaying univariate association with a significance threshold of *p* = 0.20 with a chi-square test, then removing from the model non significant variables (threshold of *p* = 0.05). We then tested each removed variable for their significance if added. Statistical analyses were conducted using Python (Pandas–Scipy–Statsmodel).

## 3. Context: The French Media Coverage of Clinical Research

**Figure 1:**
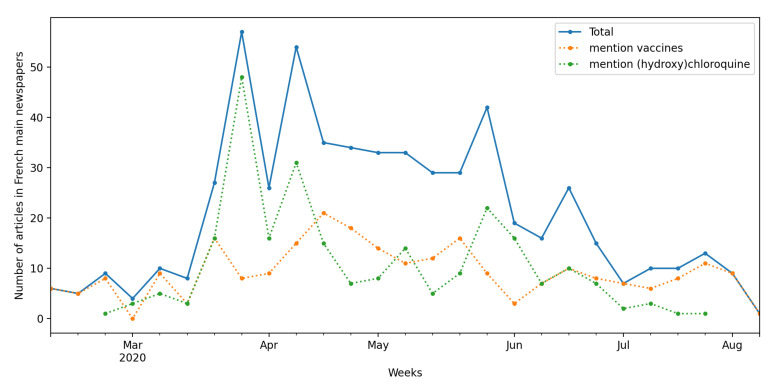
Evolution of media coverage of medical research in French national newspapers. Articles from the main French newspapers (Europress database) mentioning “clinical research” or “clinical trials”. Absence of data indicates absence of mention in articles over the period.

Since the public acknowledgement of the pandemic by French health authorities, the hope for a cure generated intense media coverage. The focus was initially on the WHO trial and the first vaccine attempts in Asia and then shifted to the various clinical trials that sought to prove the efficacy of specific drugs [34]. Media coverage increased drastically with the lockdown implemented by the French government on 17 March 2020 (Figure 1). Two main topics linked to clinical trials dominated the headlines: the controversy surrounding the efficacy of the hydroxychloroquine treatment and vaccine trials.

Media coverage of the debates on hydroxychloroquine was particularly intense. Indeed, the main protagonists in these debates made extensive use of communication channels, beyond the norms of usual scientific practice. Professor Didier Raoult, physician and researcher in infectious diseases, was very active on YouTube and on social networks through their Twitter account, and he published parts of non-peer-reviewed scientific articles directly on the website of their institute. His critics were thus prompted to occupy similar spaces: they went on TV and published articles in mainstream newspapers to explain how clinical research is organised, how evidence is established and the nuances between the different forms of trials. Former and current political representatives also took up the cause of hydroxychloroquine, with a previous Health Minister Philippe Douste-Blazy launching a petition in defense of this treatment. Polling organisations at the time even surveyed French people’s beliefs about the effectiveness of hydroxychloroquine.

The scientific controversy over the effectiveness of hydroxychloroquine unfolded in the public sphere. Commentaries focused on the findings, methodologies and biases of ongoing trials, but also on the presumed conflicts of interest of their investigators. The government published regular progress reports on the Discovery clinical trial (NCT04315948) set up by the French biomedical research institute (INSERM), and the protocol of this trial was widely criticised by its opponents.

These various controversies had an impact on clinical research itself. Most notably, recruitment in the Discovery trial slowed down enough to be covered in the mainstream news [4]. Despite the accumulation of negative results, many resources were devoted to testing hydroxychloroquine to the detriment of other candidate drugs.

Public coverage of and debate over clinical research moved beyond the search for treatment to include: the degree of transmission by children, the effectiveness of masks and the development of more effective respirators or more reliable tests. Health researchers were put in the spotlight, with high expectations both in terms of the production of knowledge on this novel virus and in terms of finding practical solutions to control and treat it.

## 4. Results

### 4.1. French Attitudes toward Clinical Research

The description of the 1003 participants is provided in Table 2.

A majority of respondents (64.8%) declared that they were at least somewhat familiar with clinical trials (Table 2), with 19.5% reporting high familiarity. Moreover, almost one out of two respondents (48.6%) declared that they knew that the clinical research process is divided into several phases. Attitudes toward clinical trials were positive overall: the average attitude scale score was 6.57 out of 10 (SD 1.67; 95% CI [6.46–6.57]) and 72.0% of respondents were classified as having positive attitudes toward clinical trials (score ≥ 6).

Respondents gave great importance to clinical research, with almost three-quarters (71.8%) disagreeing with the statement that the fight against unemployment (which has been one of the primary concerns of the French public for several years) takes precedence over the funding of clinical research (Q-B-3).

There was very strong agreement on the role played by clinical research in medical advances (Table 2, Q-A-6), and clinical trials were largely associated with access to treatments (Q-A-2 and Q-A-5). Respondents (see Figure S1) generally associated clinical trials with the production of knowledge and the development of new treatments, with a strong correlation between the question Q-A-6 “advance of medical knowledge”, Q-A-5 “access to new treatments” (0.49, *p* < 0.01) and Q-A-2 “access to alternative treatments” (0.43, *p* < 0.01).

**Figure 2:**
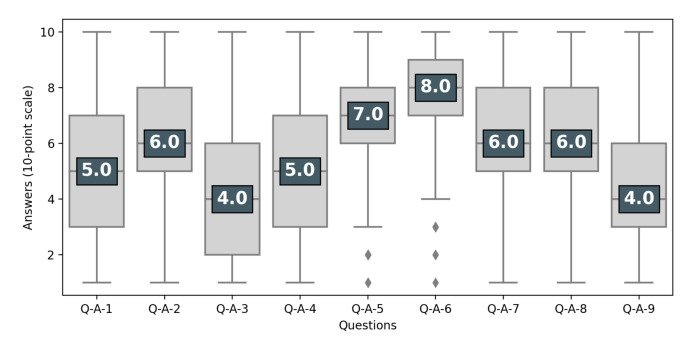
Perception of clinical trials. Respondents having at least heard of clinical trials (N = 966). Questions are presented in Table 1. The number is the median of the answers’ distribution, the box indicates the inter-quartile range (50% of answers), the bar indicates 95%.

Attitudes toward the status of participants in clinical trials were much more divided (see Figure 2), with 41% of the respondents stating that participants were only “guinea pigs” (Q-A-4). Likewise, there was considerable variation in attitudes toward whether experimental treatments are like any other treatment (Q-A-9) and toward the right of individuals to participate in clinical trials despite contrary advice from their doctor (Q-A-7).

Respondents recognized that clinical research was not just conducted by physicians (only about one-third of respondents agreed with this statement) and that the pharmaceutical industry also played an important role (68.4%). However, while they expressed high trust in doctors and scientists (93% and 89%), they expressed low trust in the industry and in politicians (26% and 18%). Three different positions toward the role of the pharmaceutical industry in clinical research were identified: respondents who did not trust the industry but acknowledged its importance for clinical research (46%), respondents who neither trusted the industry nor acknowledged its importance (28%) and respondents who trusted the industry and either acknowledged its importance (22%) or not (4%).

### 4.2. Factors Associated with Familiarity with and Attitudes toward Clinical Trials

In univariate analysis (Table 3), we observed no significant association between either familiarity or attitudes with age, health condition and COVID-19 concerns. On the contrary, we observed a significant association with education, health literacy and health information seeking-behaviour. Finally, other factors appeared associated only with familiarity (trust in industry) or only with a positive attitude (male sex; trust in doctors, scientists and politicians; lack of financial difficulties).

**Figure 3:**
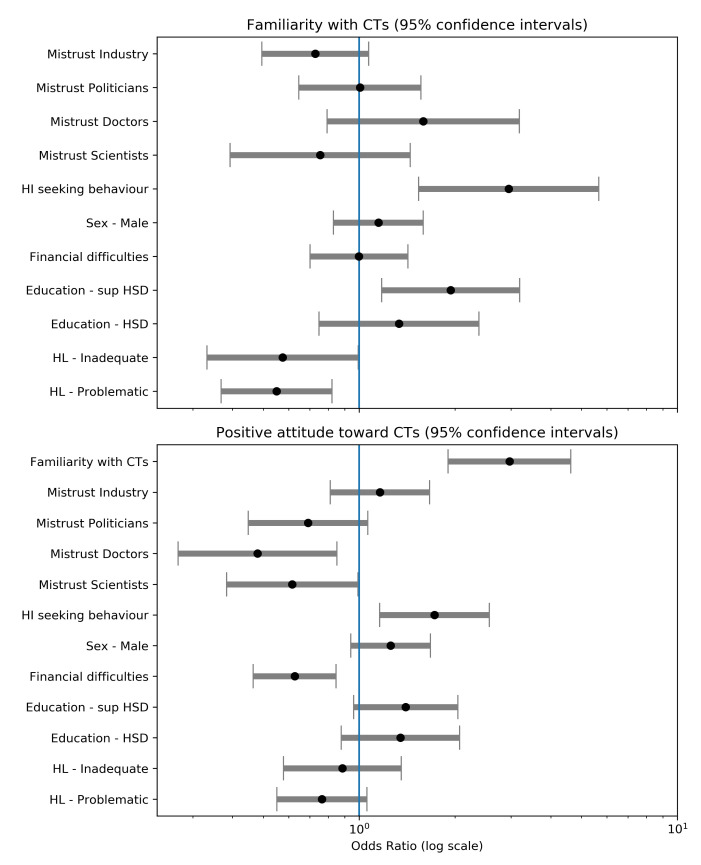
Odds ratio from logistic regressions on familiarity and attitude Abbreviations: HSD, High School Degree; HL, Health Literacy; HI, Health Information.

To control the effect of those correlating factors, we fitted two binomial logistic regressions retaining only significant associations: sex, education, health literacy, health information-seeking behaviors and trust in institutions (Figure 3, Table S1). McFadden’s pseudo-$R^{2}$ are, respectively, 4% and 7%. Educational attainment above the high school diploma (compared to below, odds ratio, OR 1.94 [1.18–3.19]) and having health-information seeking behaviors (compared to not having them, OR 2.95 [1.54–5.66]) were associated with high familiarity, whereas problematic or inadequate health literacy (compared to adequate, OR 0.55 [0.37–0.82]) was associated with low familiarity. No association was found between familiarity and other factors, especially financial difficulties or trust, even if trust in industry is at the margin of a *p* = 0.1 threshold (OR 0.73 [0.49–1.07]).

Having a positive attitude was strongly associated with reporting good familiarity with clinical trials (compared to being unfamiliar, OR 2.97 [1.90–4.63]) and having a health information-seeking behavior (compared to reporting not having, OR 1.72 [1.16–2.56]). Lack of trust in doctors or scientists almost halved the probability of having a positive attitudes toward clinical trials (respectively, 0.48 [0.27–0.85] and 0.62 [0.38–0.99] compared to reporting trust). There was a similar effect for lack of trust in politicians (OR 0.69 [0.45–1.06]) but only significant at the *p* = 0.10 threshold. Finally, reporting financial difficulties was associated with a lower likelihood of reporting positive attitudes (OR 0.63 [0.46–0.85]). There was no effect of trust in industry and health literacy.

## 5. Discussion

### Attitudes toward Clinical Research in a Context of Strong Mistrust of the Pharmaceutical Industry and Politicians

While the fight against cancer and other epidemics such as HIV have given some public visibility to clinical research, the sheer scale of the COVID-19 pandemic has put this issue in the media spotlight at a global scale. This survey carried out at the end of the first wave of the COVID-19 epidemic in France revealed a shared familiarity with and positive attitudes toward clinical research among the French public. Moreover, the perceptions of clinical trials as potential treatment and as an activity aiming to produce knowledge are largely intertwined. Yet, in the midst of such intense media coverage and a shared awareness of the importance of clinical research, public perceptions include also a strong mistrust of the pharmaceutical industry and politicians. During the first months of the COVID-19 pandemic, many public figures denounced the influence of pharmaceutical companies in the medical world, for instance during the controversy on the efficiency of hydroxychloroquine [29]. Though aspects of this critique may well be a reasonable response given the influence of corporate interests in research and health care [35], this may have harmful consequences on epidemic management by limiting enrollment in clinical trials, leading to underpowered results [36], and reinforcing conspiracy theories surrounding an all-powerful pharmaceutical lobby [37,38].

To our knowledge, this is the first study in the international litterature to provide conjointly a measure of familiarity with and attitudes toward clinical trials in recent decades. The vast majority of past studies have focused on direct users of clinical trials, i.e., patients and physicians [21,22,23]. However, as the authority of science has been challenged in the past decades and health crises have politicized medical innovations, there is a need to better understand how the public conceives of medical research. In this study, a significant proportion of the French population claimed some familiarity with clinical trials. This proportion was surprisingly high for such a specialised topic. The French media, especially the most mainstream news outlets, likely contributed to raising awareness of clinical trials research, as in the debate over the efficacy of hydroxychloroquine [39]. These results echo those of other international studies reporting the prevalence of positive attitudes toward clinical trials [20,25]. The results also point out that, beyond this overall agreement, French people are divided regarding the benefits one could expect from clinical trials.

Familiarity with and attitudes toward clinical trials in the specific context of France were found to depend on different factors. While we expected health literacy and education level to be associated with familiarity with clinical trials, we were surprised to find no effect of economic disadvantage or age on familiarity [24]. As positive attitudes were strongly associated with familiarity with clinical trials, we can expect that a main driver of attitudes is the proximity with health and science information. In the same way, health information seeking-behavior was associated with both familiarity and positive attitudes. This derives from the fact that, regardless of familiarity, such behavior is positively associated with attitudes perhaps through a higher interest in medical innovation. Conversely, mistrust of doctors, scientists and potentially politicians (even if the effect is only significant at the *p* = 0.1 threshold) tended to affect negatively attitudes toward clinical trials, suggesting that the relationship to institutional authority has an impact on the perception of clinical research [40]. This effect of trust in authority figures is an important factor to consider for health communication, for the organisation of clinical research and for reporting research advances. Hence, enrollment of patients in clinical trials would be impacted by how research is framed politically in the public sphere. Not surprisingly the strongest association was with trust in doctors, as trial enrollment finally relies on a proposal made by a doctor. It has been argued that communication that promotes trust and confidence in the doctor might be a powerful motivating influence [20,41]. We should stress that the low explained variance of the two logistic suggest the need to explore further factors explaining familiarity and attitude toward clinical trials.

One of the most intriguing findings of this study is that positive attitudes toward clinical research exist side by side with a strong distancing from the pharmaceutical industry. While respondents acknowledged the diversity of actors involved in clinical research, they also expressed a strong mistrust toward the pharmaceutical industry, with almost a third stating that the latter does not play an important role in medical innovation. This reflects the perception of a clear division between public and private research and an enduring suspicion toward the private sector. There is no question that pharmaceutical industries shape large parts of medical research [15,42,43]. Nevertheless, this division is alarming in a context where the recent reforms proposed by the French government have been severely criticised for their entrepreneurial orientation [44,45]. The discrepancy between this perception and the reality of the pharmaceutical industry’s role in clinical research, already pointed out [13], could be problematic in this pandemic and future health crises.

Another finding relates to the effect of economic disadvantage on negative attitudes toward clinical trials. While there appeared to be little correlation between economic disadvantage and trust toward politicians, doctors and scientists, our findings seem to identify a specific effect of financial difficulty on positive attitudes toward clinical trial. One possible reason for this might include the different experience of care of people with economic disadvantage, a lack of knowledge that clinical trial participation is covered by universal health care in France, or a fear of hidden costs related to participation [46]. This finding and the multiple possible hypotheses should be explored in future research, because a different perception of the relation between research and care [47] can lead to consequences in clinical trial enrollment. National differences are likely to impact attitudes toward clinical trials. If they can be seen as an alternative of unaffordable care in the US, framing them as a health option, it is not the case in France where the social security foster access to standard therapies. There is a need to further explore how medical research is framed by health expectations. We can expect important differences between diseases, as in the use of clinical research as subsidiary care option in cancer [11] and other life threatening diseases [48]. Given this effect of economic disadvantage and other recent research on inequities in COVID outcomes, it will be important to further study inequities related to clinical trials and health care experiences. Higher education and health literacy level were not significantly associated with attitudes after adjustment for familiarity with clinical trials, but they were strongly associated with familiarity. A first step to promote trial participation is probably the struggle against information inequalities.

During the COVID-19 pandemic, the media presented a more comprehensive picture of clinical research, while commenting extensively on the links between public and private sectors in this domain. Indeed, the existence of conflicts of interests has long been an important critique invoked to explain relations between physicians and industry [49]. This understanding may also have the potential to weaken trust in clinical research and in science and medicine more generally. In addition to undermining public authority for science, this mistrust may fuel public criticism of health policy as well as reticence to follow recommendations for preventive health behaviors. Hence, vaccine hesitancy has been shown to build on such distrust, especially in France [27,50]. Our survey opened new questions regarding public perceptions of medical research, especially regarding how the divided attitudes toward actors involved in medical research impact the way it is perceived, and how COVID-19 media coverage of medical innovations will impact public perception of medical research into the future.

## 6. Limitations

This study has several limitations. It was designed initially as a survey of attitudes toward clinical research unrelated to the coronavirus epidemic. However, additional questions were developed, piloted and included to account for attitudes toward clinical research on COVID-19. This exploratory aim allows the study to raise new questions that should be explored more thoroughly in future studies. Especially, the next step will be to propose a more robust model to specify explicative factors. The low pseudo-$R^{2}$ of the two logistic models limit them to explore associated factors. The second limitation is the non-probabilistic survey. It is nevertheless nationally representative regarding age group, gender, region and population density of residence through quota sampling. This limits the statistical representativeness of this study and points to the importance of confirming these results in other surveys. Another limitation involves the low number of questions regarding the pharmaceutical industry (two items) and regarding the relationship between clinical research and society (two items). As this study aimed to provide an initial overview of perceptions of clinical research in France, questions concerning, for example, political orientation or familiarity with specific aspects of clinical research were set aside and will be important for future research. Surveys should also contain more specific questions regarding respondents’ experience of clinical trials or that of their relatives. This was not included in this survey to limit the number of items. Another improvement would be to have more information regarding personal involvement in health practices to better capture its effect on familiarity and attitude. The third and final limitation of this study is that a questionnaire cannot fully capture the diversity of perceptions of clinical research, suggesting a need for qualitative studies on this topic. In this regard, some questions (for instance, the question regarding familiarity toward the phases of clinical trials) may give rise to a “desirability bias” and future qualitative research could increase reliability.

## 7. Conclusions

The promise of medical innovation plays a crucial role in the management of epidemics. Public attitudes toward clinical research affect both the organisation of clinical trials and trust in health policy. In a context in which medical, scientific and political authority is increasingly challenged, it is important to more completely understand what people know about clinical research and how the latter is discussed in the public sphere.

Even if scientific research is international, health issues and experiences are deeply embedded in national trends. Hence, in the French context, mistrust of the pharmaceutical industry remains strong. Fears that industry interference may work against the public good are legitimate, and researchers have widely demonstrated the damaging impact of such interference on clinical research, for instance by the tobacco industry [51]. Such negative perceptions of the pharmaceutical industry and its role in medical research and health care need to be further explored as contemporary biomedical innovation depends on collaboration between the public and private sectors. The perception of an enduring division between a “pure” public research and a “biased” private interest does not fit current medical innovation organization and may make public debates more contentious and prevent effective reforms to strengthen the public sector. While ongoing political reforms threaten public research autonomy and funding, the public needs to better understand the current practices of clinical research in order to undertake constructive debates.

We suggest three potential outcomes of the present research as well as the need for further research. Given that the findings indicate that the public is divided on the role of the pharmaceutical industry in medical innovation and that this role is highly controversial, health national authorities worlwide should make sure via investigation and policy-making that the interests of pharmaceutical corporations do not supersede nor detract from the public good, and safeguards already in place or implemented to protect the public good in the current system of medical innovation should be highly publicized to prevent destructive polemics when they arise. The French case suggests that media coverage of science and scientific involvement in social media and the public sphere are an incredibly important force affecting public attitudes toward health practices and policies. While biomedical research is frequently invoked to illustrate scientific progress, it tends to become highly politized [5]. Finally, there is little doubt that the COVID-19 pandemic has transformed public attitudes toward medical research. Accelerating research processes and ways science is communicated, fostering expectations followed by disappointment, and unveiling the interdependence between states, pharmaceutical companies, physicians and scientists, this new disease is contributing to a new public frame of what medical research is that will surely impact other sectors.

## Figures and Tables

**Table 1:** Questions regarding attitudes toward clinical trials.

	**Questions on Clinical Trials**	**1 = Disagree Completely; 10 = Agree Completely**
Q-A-1	Clinical trials are only useful as a last resort-after trying all available treatments	10-point scale
Q-A-2	Clinical trials offer an alternative to a treatment that you wish to avoid (invasive surgery, chemotherapy, etc.)	10-point scale
Q-A-3	Clinical trials are only suitable for people with a life-threatening condition	10-point scale
Q-A-4	Participants in clinical trials are only “guinea pigs”	10-point scale
Q-A-5	Clinical trials give people hope by giving them access to new treatments that they could not get otherwise	10-point scale
Q-A-6	Individuals who participate in research help advance medical knowledge and treatments for other sick people	10-point scale
Q-A-7	Sick people should have the right to test new drugs if they wish to do so, even if doctors disagree	10-point scale
Q-A-8	Receiving experimental treatment is a great opportunity	10-point scale
Q-A-9	Experimental treatments are like any other treatment	10-point scale
	**Questions on Medical Research**	**1 = Agree Completely; 4 = Disagree Completely**
Q-B-1	Industry plays an important role in clinical research	4-point scale
Q-B-2	Medical research is done only by doctors	4-point scale
Q-B-3	Fighting unemployment is more important than funding medical research	4-point scale
Q-B-4	Citizens must be able to give their opinion on public research choices	4-point scale
Q-B-5	Priority should be given to new diseases	4-point scale
Q-B-6	The way research is done on cancer/covid is specific compared to other diseases	4-point scale

**Table 2:** Sample description and attitudes toward medical research.

Variables		Weighted Frequency	Proportion (%)
Sex	female	515.5	51.4
	male	487.5	48.6
Age	[18–35]	306.9	30.6
	[35–45]	189.6	18.9
	[45–55]	195.6	19.5
	[55–65]	182.5	18.2
	[65–75]	128.4	12.8
Education	1-Below HSD	169.8	16.9
	2-HSD	240.1	23.9
	3-Above HSD	593.1	59.1
Financial difficulties	No	643.0	64.1
	Yes	360.0	35.9
Health condition	1-Good	649.2	64.7
	2-Average	281.3	28.0
	3-Bad	72.5	7.2
HI seeking behaviour	No	136.1	13.6
	Yes	866.9	86.4
Concerns about COVID19	No	54.5	5.4
	Some	683.6	68.2
	Yes	264.9	26.4
Job in health sector	No	827.4	82.5
	Yes	175.6	17.5
Health literacy	1-Adequate	604.8	60.3
	2-Problematic	263.6	26.3
	3-Inadequate	134.6	13.4
Familiarity with CTs	1-Never heard of CTs	37.6	3.7
	2-Only know the term, CTs	315.1	31.4
	3-Somewhat familiar with what CTs are	454.6	45.3
	4-Very familiar	149.2	14.9
	5-Extremely familiar	46.5	4.6
Know that CTs are divided in phases	NA	37.6	3.7
	No	479.4	47.8
	Yes	486.0	48.5
Attitudes toward CTs	Negative < 5	78.7	8.2
	Neutral = 5	191.4	19.8
	Positive > 5	695.3	72.0
Trust in doctors	1-Yes	933.8	93.1
	2-No	69.2	6.9
Trust in researchers	1-Yes	896.6	89.4
	2-No	106.4	10.6
Trust in the industry	1-Yes	258.1	25.7
	2-No	744.9	74.3
Trust in politicans	1-Yes	178.4	17.8
	2-No	824.6	82.2
Q-B-1 (importance of industry)	Agree	685.3	68.3
	Disagree	317.7	31.7
Q-B-2 (only doctors do medical research)	Agree	293.5	29.3
	Disagree	709.5	70.7
Q-B-3 (priority of fighting unemployement)	Agree	282.6	28.2
	Disagree	720.4	71.8
Q-B-4 (importance of citizens’ opinion)	Agree	747.1	74.5
	Disagree	255.9	25.5
Q-B-5 (prioritize new diseases)	Agree	416.1	41.5
	Disagree	586.9	58.5
Q-B-6-cancer (cancer research is specific)	Agree	200.6	40.0
	Disagree	301.0	60.0
Q-B-6-corona (covid research is specific)	Agree	253.7	50.6
	Disagree	247.7	49.4

Frequencies have decimal due to weighted quotas. Abbreviations: CTs, Clinical Trials; NA, Not Answering; HSD, High School Diploma.

**Table 3:** Univariate associations for good familiarity and positive attitudes.

		Good Familiarity	*p*	Positive Attitude	*p*
Sex	female	93.4 (18.1%)	0.2897	342.1 (66.4%)	0.0432
	male	102.2 (21.0%)		353.2 (72.5%)	
Age	[0–25]	25.4 (19.5%)	0.783	85.1 (65.3%)	0.115
	[25–45]	69.2 (18.9%)		249.9 (68.3%)	
	[45–65]	79.2 (20.9%)		260.0 (68.7%)	
	[65+]	21.9 (17.1%)		100.3 (78.1%)	
Education	1-Below HSD	21.7 (12.8%)	0.0025	102.3 (60.2%)	0.0043
	2-HSD	37.3 (15.5%)		160.5 (66.8%)	
	3-Above HSD	136.6 (23.0%)		432.5 (72.9%)	
Financial difficulties	No	133.0 (20.7%)	0.2422	476.1 (74.0%)	<0.0001
	Yes	62.7 (17.4%)		219.1 (60.9%)	
Health litteracy	1-Adequate	139.8 (23.1%)	0.0018	439.8 (72.7%)	0.0151
	2-Problematic	37.2 (14.1%)		171.1 (64.9%)	
	3-Inadequate	18.7 (13.9%)		84.5 (62.8%)	
HI seeking behaviour	No	11.3 (8.3%)	0.0006	76.3 (56.1%)	0.0005
	Yes	184.4 (21.3%)		619.0 (71.4%)	
Health condition	1-Good	126.4 (19.5%)	0.768	452.6 (69.7%)	0.4605
	2-Average	52.9 (18.8%)		188.8 (67.1%)	
	3-Bad	16.4 (22.6%)		53.9 (74.3%)	
COVID19 concerns	No	14.2 (26.1%)	0.2999	34.2 (62.8%)	0.5451
	Some	125.8 (18.4%)		477.8 (69.9%)	
	Yes	55.7 (21.0%)		183.3 (69.2%)	
Trust in scientists	1-Yes	180.6 (20.1%)	0.1818	641.0 (71.5%)	<0.0001
2-No	15.1 (14.2%)	54.3 (51.0%)			
Trust in doctors	1-Yes	181.6 (19.4%)	0.9754	662.5 (71.0%)	0.0001
2-No	14.1 (20.4%)	32.7 (47.3%)			
Trust in politicians	1-Yes	40.8 (22.9%)	0.2525	138.7 (77.7%)	0.0093
2-No	154.9 (18.8%)	556.6 (67.5%)			
Trust in the industry	1-Yes	62.5 (24.2%)	0.0339	185.2 (71.8%)	0.3647
2-No	133.2 (17.9%)	510.0 (68.5%)			

For each variable, we computed a contingency table (total count and proportion with weights) and a $\chi^{2}$ test. Abbreviations: HI, Health Information; HSD, High School Diploma.

## Data Availability

The data that support the findings of this study are available on request from the corresponding author, E.S.

## Supplementary Materials

The following are available at https://www.mdpi.com/1660-4601/18/5/2611/s1, Figure S1: Correlation Heatmap for Q-A questions (Pearsonś correlation), Table S1: Logistic regressions for good familiary and positive attitude.
