# Supplementary material for: French Public Familiarity and Attitudes toward Clinical Research during the COVID-19 Pandemic
Source: Int J Environ Res Public Health. 2021 Mar 5;18(5):2611. doi: 10.3390/ijerph18052611 (PMC7967331; doi:10.3390/ijerph18052611)
Supplement: Supplementary file 1 [file ijerph-18-02611-s001.pdf]

Figure 1: Supplementary Figure S1 : Correlation Heatmap for Q-A questions (Pearson's correlation)

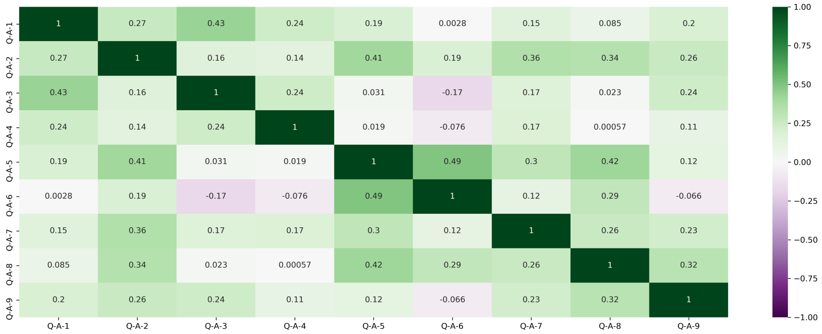

Figure 2: Supplementary Table S2 : Logistic regressions for Good familiarity (GF) and Positive attitude (PA)

	GF OR [CI 95%]	GF p	PA OR [CI 95%]	PA p	GF Wald	p	df	PA Wald	p	df
Intercept	0.08 [0.03-0.19]	¡0.000***	1.49 [0.81-2.72]	0.198						
Sex - Woman					0.704	0.401	1	2.374	0.123	1
Man	1.15 [0.83-1.59]	0.401	1.25 [0.94-1.67]	0.123						
Education - inf HSD					8.301	0.016	2	3.168	0.205	2
HSD	1.33 [0.75-2.38]	0.329	1.35 [0.88-2.07]	0.172						
sup HSD	1.94 [1.18-3.19]	0.009***	1.40 [0.96-2.04]	0.080*						
Financial difficulties - No					¡0.000	0.995	1	9.372	0.002	1
Yes	1.00 [0.70-1.42]	0.995	0.63 [0.46-0.85]	0.002***						
Health litteracy - Adequate					10.656	0.005	2	2.648	0.266	2
Problematic	0.55 [0.37-0.82]	0.003***	0.76 [0.55-1.06]	0.105						
Inadequate	0.57 [0.33-0.99]	0.047**	0.89 [0.58-1.36]	0.580						
HI seeking behaviour - No					10.612	0.001	1	7.226	0.007	1
Yes	2.95 [1.54-5.66]	0.001***	1.72 [1.16-2.56]	0.007***						
Doctors - Trust					1.701	0.192	1	6.302	0.012	1
No trust	1.59 [0.79-3.19]	0.192	0.48 [0.27-0.85]	0.012**						
Scientists - Trust					0.718	0.397	1	3.989	0.046	1
No trust	0.75 [0.39-1.45]	0.397	0.62 [0.38-0.99]	0.046**						
Politicians - Trust					0.001	0.981	1	2.822	0.093	1
No trust	1.00 [0.65-1.56]	0.981	0.69 [0.45-1.06]	0.093*						
Industrials - Trust					2.601	0.107	1	0.666	0.414	1
No trust	0.73 [0.49-1.07]	0.107	1.16 [0.81-1.66]	0.414						
CT familiarity								23.04	0	1
No CT familiarity			2.97 [1.90-4.63]	¡0.000***						

Figure 3: *

Abbreviations : GF, Good familiarity ; PA, Positive attitude ; OR, Odds Ratio ; p, p value; CI, Confidence Interval ; Wald, Wald statistics ; df, degrees of freedoom ; HSD, High School Degree ; CT, Clinical Trial.
